# Modeling the Niche Suitability of Grey Crowned Crane (*Balearica regulorum*) to Inform Conservation Strategies in Kenya

**DOI:** 10.1002/ece3.73446

**Published:** 2026-04-09

**Authors:** Carolyne M. Wachu, Benson M. Mwangi, James Jumbe, Wanyoike Wamiti, Denis Kiptoo

**Affiliations:** ^1^ Department of Zoological Sciences Kenyatta University Nairobi Kenya; ^2^ Department of Physical and Biological Sciences Murang'a University of Technology Murang'a Kenya; ^3^ International Crane Foundation (ICF) Baraboo Wisconsin USA; ^4^ Regional Centre for Mapping of Resources for Development Nairobi Kenya

**Keywords:** Grey Crowned Crane conservation, habitat suitability, species distribution models

## Abstract

The Grey Crowned Crane is listed globally as Endangered, primarily due to threats such as habitat loss, degradation, and fragmentation, as well as human encroachment, electrocution, and illegal capture. Effective conservation requires knowledge of the species' current and future spatial distribution under changing climatic conditions. This study addresses these knowledge gaps by assessing the climate suitability and spatial distribution of the species in Kenya under current and future climate scenarios. Using occurrence data from the Global Biodiversity Information Facility and climatic variables from WorldClim, species distribution models were developed to evaluate the species' suitable habitats. The influence of climatic predictors on species distribution was assessed under two climate change scenarios (SSP2‐4.5 and SSP5‐8.5) for the period 2020–2040. An ensemble modeling approach combining Generalized Linear Models, Generalized Additive Models, and Random Forest algorithms was used to predict suitable crane habitats. The distribution map was overlaid with protected area boundaries to assess the coverage of habitat protection. Results indicate that suitable habitats are largely concentrated in western and southwestern Kenya, with distribution strongly influenced by temperature and precipitation. The predicted reduction in suitable area, about 49.5% under SSP2‐4.5 and 47.5% under SSP5‐8.5, highlights the vulnerability of the species to changing climatic conditions. Projections under the two climate change scenarios revealed a potential contraction in the suitable range, indicating possible adverse impacts of climate change. These findings provide insights into the climatic drivers of Grey Crowned Crane distribution and offer a basis for informing conservation planning and habitat management under future climate change.

## Graphical abstract

This study used ensemble species distribution models to assess the current and future climatic suitability of habitats for the Grey Crowned Crane (*Balearica regulorum*) in Kenya. Using occurrence data and bioclimatic variables, we identified key climatic drivers of habitat suitability and projected substantial contractions in suitable habitats under future climate scenarios. The results highlight the vulnerability of the species to climate change and emphasize the need for climate‐informed conservation strategies, particularly in landscapes outside formal protected areas.

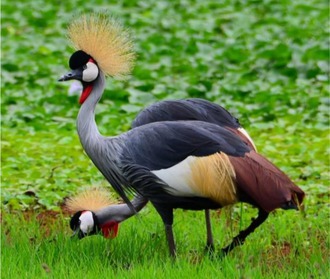

## 1 Introduction

**FIGURE 1:**
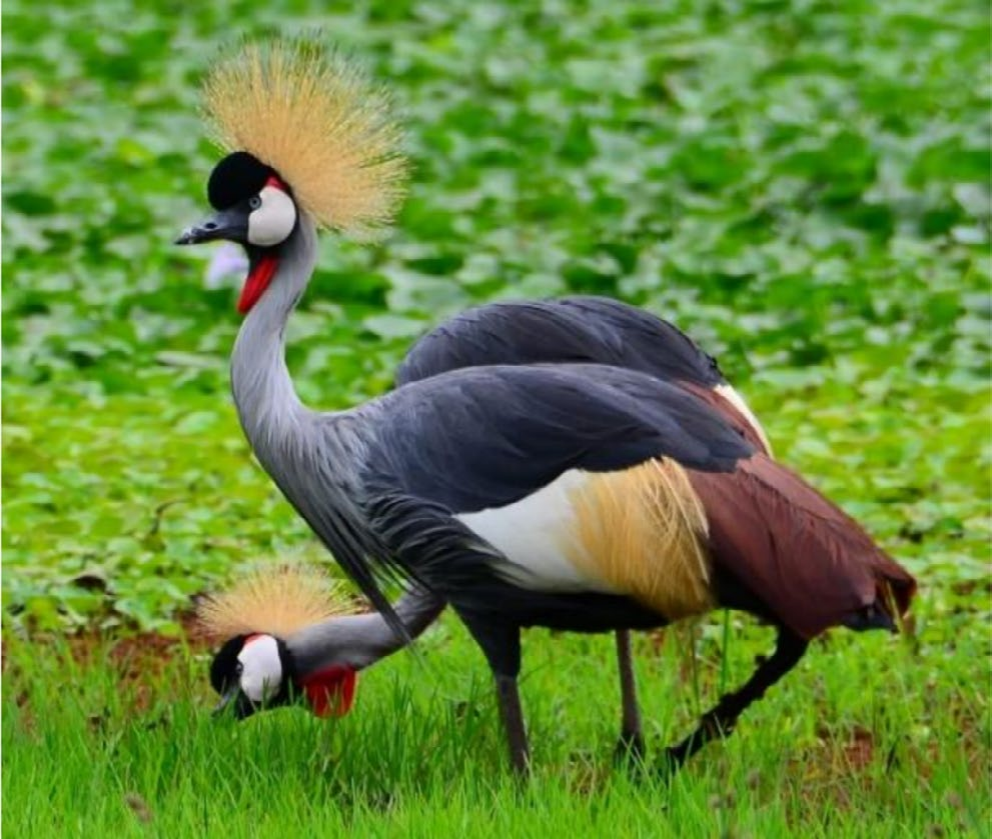
Grey Crowned Crane observed in Ecoscape Sanctuary, Naivasha.

The Grey Crowned Crane ( *Balearica regulorum* ) (Figure 1) is listed as an Endangered species on the IUCN Red List of Threatened Species (BirdLife International 2025). Eastern Africa remains a critical stronghold, supporting most of the global population, particularly in Uganda and Kenya, with the latter hosting the largest numbers (Meine and Archibald 1996; BirdLife International 2020). The species, a member of the family *Gruidae*, belongs to one of the most threatened bird families globally (Morrison 2015; Fakarayi et al. 2016). Among the fifteen crane species worldwide, the Grey Crowned Crane has experienced one of the most rapid population declines (Morrison et al. 2019). Its population is rapidly decreasing across its range, primarily due to habitat loss (Beilfuss et al. 2007; Morrison et al. 2019). Other threats include electrocution, illegal removal from the wild, persecution, and climate change (Harris and Mirande 2013; Meles and Gemeda 2019; Wamiti et al. 2023).

Cranes serve as a valuable natural resource, playing a vital role in the environment as indicators of the condition of their grassland and wetland habitats (Nsengimana et al. 2019). Additionally, they contribute to foreign exchange through tourism (Olupot et al. 2010). The positive impact of the cranes extends to water purification and the establishment of recreational spaces (Patterson‐Abrolat et al. 2018). Therefore, conserving crane habitats is crucial to safeguard the species from further decline or extinction.

Understanding and protecting biodiversity is crucial for species conservation and management. Knowledge of habitat requirements and species distribution supports effective habitat protection and natural reserve management. Climate change poses significant threats to biodiversity by altering habitats, species ranges, and extinction rates (Nguyen et al. 2021). For wetland‐dependent species such as the Grey Crowned Crane, rising temperatures and changing precipitation patterns can directly affect wetland hydrology, vegetation structure, and food availability, potentially reducing habitat suitability and fragmenting populations (Parmesan and Yohe 2003; Chen et al. 2011; Wamiti et al. 2023). These environmental changes highlight the importance of assessing the ecological niche and distribution of the Grey Crowned Crane to inform species‐specific conservation planning and habitat management.

From an ecological perspective, the distribution of species is largely determined by their ecological niche, which represents the range of environmental conditions under which a species can survive and reproduce (Soberón 2007). According to ecological niche theory, environmental factors such as temperature, precipitation, and habitat characteristics influence the spatial distribution of species by defining the environmental conditions that support their persistence (Elith and Leathwick 2009; Peterson et al. 2011). Consequently, understanding these environmental drivers is essential for predicting species responses to environmental change (Ehrlén and Morris 2015).

Species Distribution Modeling (SDM) is a valuable tool for analyzing and predicting species distribution under current and future environmental conditions (Alsamadisi et al. 2020). By linking environmental factors with species presence or abundance, SDMs provide insights into the potential impacts of environmental changes (Kaky and Gilbert 2019). These models are widely used to evaluate habitat suitability, forecast suitable regions, and guide conservation strategies, particularly for endangered species (Urban 2015). SDMs also help predict changes in species distributions under various climate scenarios, making them essential for addressing biodiversity challenges.

Despite the ecological and conservation importance of the Grey Crowned Crane, comprehensive analyses of its ecological niche and spatial distribution in Kenya remain limited. In particular, there is insufficient understanding of how climatic variables influence its distribution patterns across the country. Addressing this knowledge gap is essential for informing conservation planning and habitat management for the species.

In light of this context, the present study seeks to enhance knowledge of the biogeographical patterns of the Grey Crowned Crane in Kenya. The study specifically aimed to: (i) examine the species' ecological niche and spatial distribution within the country, (ii) assess the relative influence of climatic factors on its distribution, and (iii) offer informed recommendations for the conservation and management of its habitats. Based on ecological niche theory and the influence of climatic drivers on species distributions, we hypothesize that:

- The spatial distribution of the Grey Crowned Crane in Kenya is strongly influenced by climatic variables.
- Suitable habitats for the species are spatially heterogeneous and concentrated in specific ecological regions.
- Identifying the climatic drivers of habitat suitability can support improved conservation planning and habitat protection for the species.

## 2 Materials and Methods

### 2.1 Study Area

Kenya is approximately 580,367 km^2^ in size, situated between latitudes 5°40′ N and 4°04′ S and longitudes 33°60′ E and 41°45′ E (Kiunga 2015). The country experiences a predominantly tropical climate, with notable regional variations. Coastal regions are typically hot and humid, while the central highlands are cooler and temperate. The northern and northeastern areas are predominantly arid. Rainfall is bimodal, with the long rains occurring from March to May and short rains from October to December (Ayugi et al. 2016). Average temperatures range from 15°C to 35°C, depending on the altitude and location (Downing et al. 2023).

The country's terrain is varied, featuring coastal plains, plateaus, savannahs, the Rift Valley, and highland regions. Kenya is rich in conservation areas, hosting 22 national parks covering about 29,357 km^2^, 28 national reserves covering 18,042 km^2^, and more than 160 community and private conservancies totaling approximately 36,300 km^2^, in addition to 68 Important Bird and Biodiversity Areas (Ngila et al. 2023).

### 2.2 Conceptual Framework and Analytical Approach of the Study

Understanding the spatial distribution of species requires identifying the environmental factors that determine habitat suitability and predicting how these relationships may change under future climatic conditions. Species Distribution Models (SDMs) provide a framework for linking species occurrence data with environmental predictors to estimate ecological niches and project potential distributions across geographic space (Elith and Leathwick 2009; Peterson et al. 2011).

In this study, a multi‐step analytical framework was applied to investigate the ecological niche and potential distribution of the Grey Crowned Crane in Kenya. First, species occurrence records were compiled and cleaned to ensure spatial accuracy and reduce sampling bias. Second, climatic variables representing current environmental conditions were obtained and screened for multicollinearity to identify the most relevant predictors influencing the species' distribution. Third, multiple SDM algorithms were implemented within an ensemble modeling framework to estimate current habitat suitability and improve predictive robustness.

Finally, the calibrated models were used to project potential future distributions under alternative climate change scenarios. The resulting suitability maps were reclassified into habitat suitability categories and spatially compared with protected areas in Kenya to assess the extent to which suitable habitats are represented within the current conservation network. This analytical approach enables the identification of key climatic drivers of species distribution and provides insights into potential shifts in suitable habitats under future climate conditions.

### 2.3 Source and Pre‐Processing of Species Occurrence Data

A total of 67 occurrence records of the Grey Crowned Crane located within Kenya were used in the species distribution modeling analysis. The data were obtained from the Global Biodiversity Information Facility (GBIF.org 12 June 2025, GBIF occurrence download https://doi.org/10.15468/dl.ndxqdf).

To ensure the reliability and spatial accuracy of species occurrence data, the “clean coordinates” function from the “coordinateCleaner” package in R was applied (Zizka et al. 2019). This step eliminated erroneous entries such as incomplete or zero coordinates and those misaligned with the designated study area. Only records at the species level relevant to the Grey Crowned Crane were retained, following strict taxonomic validation procedures (Verbitsky et al. 2025).

To reduce spatial autocorrelation and sampling bias, which could otherwise result in model overfitting, a spatial thinning approach was applied using the thin function from the *SpThin* package (Aiello‐Lammens et al. 2015). A thinning distance of 40 km was used to limit overrepresentation from heavily sampled areas (Boria et al. 2014). After the data cleaning process, 62 occurrence records remained and were used to develop the Species Distribution Models (SDMs), while five records were excluded due to quality concerns.

### 2.4 Source and Pre‐Processing of Environmental Modeling Data

Nineteen bioclimatic variables (Table **1**) representing current climatic conditions, with a spatial resolution of 30 arc‐seconds (approximately 1 km^2^), were downloaded from the WorldClim database (version 2.1; Fick and Hijmans 2017; https://www.worldclim.org/data/worldclim21.html). To ensure that the analysis focused only on environmental conditions within the study area, the bioclimatic predictor variables were masked to the Kenyan national boundary before model development, ensuring that model calibration and predictions were restricted to the defined study area. Cells outside Kenya were excluded entirely, so potentially suitable habitat in neighboring countries was not classified as absence.

**TABLE 1:** Bioclimatic variables considered prior to multicollinearity testing. Only a subset of these was retained for modeling after applying variance inflation factor (VIF) analysis to eliminate highly correlated predictors.

Variables	Acronym	Units	Source
Annual Mean Temperature	BIO1	Degrees Celsius (°C)	http://worldclim.org/bioclim
Mean Diurnal Range	BIO2	Degrees Celsius (°C)	http://worldclim.org/bioclim
Isothermality	BIO3	Percentage (%)	http://worldclim.org/bioclim
Temperature Seasonality	BIO4	Degrees Celsius (°C)	http://worldclim.org/bioclim
Max Temperature of Warmest Month	BIO5	Degrees Celsius (°C)	http://worldclim.org/bioclim
Min Temperature of Coldest Month	BIO6	Degrees Celsius (°C)	http://worldclim.org/bioclim
Annual Temperature Range	BIO7	Degrees Celcius (°C)	http://worldclim.org/bioclim
Mean Temperature of Wettest Quarter	BIO8	Degree Celsius (°C)	http://worldclim.org/bioclim
Mean Temperature of Driest Quarter	BIO9	Degree Celsius (°C)	http://worldclim.org/bioclim
Mean Temperature of Warmest Quarter	BIO10	Degree Celsius (°C)	http://worldclim.org/bioclim
Mean Temperature of Coldest Quarter	BIO11	Degree Celsius (°C)	http://worldclim.org/bioclim
Annual Precipitation	BIO12	Millimeters (mm)	http://worldclim.org/bioclim
Precipitation of the wettest month	BIO13	Millimeters (mm)	http://worldclim.org/bioclim
Precipitation of the Driest month	BIO14	Millimeters (mm)	http://worldclim.org/bioclim
Precipitation Seasonality	BIO15	(Coefficient of variance (%))	http://worldclim.org/bioclim
Precipitation of the Wettest Quarter	BIO16	Millimeters (mm)	http://worldclim.org/bioclim
Precipitation of Driest Quarter	BIO17	Millimeters (mm)	http://worldclim.org/bioclim
Precipitation of the warmest Quarter	BIO18	Millimeters (mm)	http://worldclim.org/bioclim
Precipitation of the coldest Quarter	BIO19	Millimeters (mm)	http://worldclim.org/bioclim

To isolate the effects of climate change on potential species distributions, only climatic variables were used as predictors. While factors such as topography and land cover are important determinants of habitat suitability at finer scales, they were excluded from this model to explicitly focus on the species' climatic niche and its potential shifts under future scenarios (following Sutton et al. 2020). Although multiple environmental components can influence species distribution models, future projections are best informed by variables derived from climate models. Predicting habitat alterations by the year 2050 remains uncertain; however, a variety of global climate models allow for the projection of future species distributions based on current climatic limitations. Additionally, at large spatial scales, climate is widely regarded as the dominant factor shaping species ranges, supporting the use of bioclimatic variables as the most appropriate predictors (Pearson and Dawson 2003).

Multicollinearity among environmental predictors can introduce bias into species distribution models by overstating the ecological importance of correlated variables (Franklin 2010). To address this issue, a stepwise variance inflation factor (VIF) analysis was performed using the *usdm* package in R (Naimi et al. 2013), whereby variables with the highest VIF values were sequentially removed until all remaining predictors fell below the selected threshold. Predictor variables with VIF values exceeding 10 were excluded to reduce redundancy among predictors (Sutton et al. 2020). Following this procedure, nine variables that best aligned with the ecological preferences of the Grey Crowned Crane were retained for model development: isothermality, temperature seasonality, annual temperature range, mean temperature of the driest quarter, precipitation of the wettest month, precipitation of the driest month, precipitation seasonality, and precipitation during both the warmest and coldest quarters.

To project future distribution patterns of the Grey Crowned Crane, we employed a General Circulation Model (GCM) based on the Hadley Centre's Global Environment Model, HadGEM3, was employed. The selected projection period spanned from 2021 to 2040. Climatic data required for these projections were obtained from the WorldClim database (version 2.1; Fick and Hijmans 2017). To account for varying global socioeconomic trajectories, two Shared Socioeconomic Pathways (SSPs) were used, which represent different levels of climate change mitigation and adaptation challenges. The numerical values associated with the SSPs represent the approximate radiative forcing levels (W/m^2^) projected by the year 2100. Specifically, SSP2‐4.5 reflects a moderate development pathway (middle‐of‐the‐road development), with an approximate radiative forcing of approximately 4.5 W/m^2^, while SSP5‐8.5 represents a high‐emission scenario driven by fossil‐fuel‐intensive growth (fossil‐fueled development) with an approximate radiative forcing of approximately 8.5 W/m^2^.

### 2.5 Species Distribution Models

The respective strengths and limitations of various species distribution modeling approaches, including regression‐based and machine learning techniques, were considered. Three modeling algorithms, Generalized Additive Models (GAMs), Generalized Linear Models (GLMs), and Random Forests (RF), were implemented to harness the complementary strengths of different modeling techniques and to reduce the uncertainty inherent in relying on a single algorithm. These models were implemented within an ensemble forecasting framework available in the *biomod2* package (version 4.2–4) in R (Thuiller et al. 2009; Smeraldo et al. 2020; Chepkirui et al. 2024). The GLMs were calibrated with a binomial link function, setting the maximum interaction level to one. GAMs also employed a binomial link function, while RF models were constructed with 750 trees, sampling half of the available predictors at each node split (Thuiller et al. 2009).

To complement the 67 occurrence records, 10,000 pseudo‐absence points were generated randomly across Kenya to represent available environmental conditions. The points were included in all models to reduce bias from presence‐only data and improve the estimation of habitat suitability (Elith and Leathwick 2009).

Consistent with prior studies utilizing SDMs, the occurrence and background data were randomly split into two subsets, 70% for model calibration and 30% for model evaluation (Smeraldo et al. 2020). The data splitting procedure was repeated twice, and the resulting evaluation metrics were averaged across both iterations. In total, 30 SDMs were produced, comprising three algorithms, each run five times with two replicates for model evaluation.

### 2.6 Model Performance Assessment

Model performance was evaluated using the Area Under the Receiver Operating Characteristic Curve (AUC), True Skill Statistic (TSS), sensitivity, and specificity (Butler and Ford 2017). The AUC, derived from the receiver operating characteristic (ROC) curve that plots sensitivity against specificity, was used to assess the model's ability to distinguish between suitable and unsuitable habitats. AUC values greater than 0.9 were considered excellent, those between 0.7 and 0.9 good, and values below 0.7 uninformative (Franklin 2010). Models with AUC scores below 0.7 were excluded from further analyses. The TSS provided an additional measure of model accuracy by accounting for both omission and commission errors, with values below 0.4 considered poor, 0.4–0.6 fair, 0.6–0.8 good, and above 0.8 excellent (Rew et al. 2020).

For ensemble modeling, individual model projections were weighted according to their AUC scores to improve predictive reliability (Marmion et al. 2008). The relative contribution of each predictor variable was derived from ensemble outputs using functionality available in the *biomod2* package (Huang et al. 2023). Ensemble projections were generated at approximately 1 km spatial resolution for both current and future climatic scenarios to assess spatiotemporal habitat dynamics. The BIOMOD function was then applied to compare potential range sizes between current and future projections, allowing evaluation of possible habitat shifts under changing environmental conditions.

### 2.7 Reclassification of Habitat Suitability Scores

To assess habitat suitability and delineate the current geographic distribution of the Grey Crowned Crane, outputs from the ensemble models were reclassified into standardized suitability classes. The continuous suitability scores, ranging from 0 (unsuitable) to 1 (highly suitable), were categorized into five discrete classes using the natural breaks method (Jenks) (Table 2), following the approach of Singh et al. (2021) and Khan et al. (2022).

**TABLE 2:** Reclassification of habitat suitability scores into discrete suitability categories.

Category	Suitability range	Description
Highly suitable	> 0.8	Land with optimal environmental conditions that fully support the species
Suitable	0.6–0.8	Land with minor climatic limitations is favorable for survival
Moderately suitable	0.4–0.6	Land with moderate climatic constraints that limit optimal conditions
Marginally suitable	0.2–0.4	Land with significant climatic limitations that may reduce the population
Unsuitable	< 0.2	Land with severe climatic limitations is unlikely to support the species

For the purposes of estimating total suitable habitat, all cells classified as marginally suitable, moderately suitable, suitable, or highly suitable were considered suitable habitat.

### 2.8 Spatial Overlap Between Grey Crowned Crane Distribution and Protected Areas in Kenya

A shapefile of protected areas in Kenya was obtained from the World Resources Institute (https://datasets.wri.org/dataset/protected‐areas‐in‐kenya) and overlaid on the current predicted distribution map of Grey Crowned Cranes to assess the spatial overlap between suitable habitats and protected area boundaries.

## 3 Results

### 3.1 Model Performance

The ensemble model showed strong predictive performance, with Area Under the Curve (AUC) and True Skill Statistic (TSS) values of 0.908 and 0.625, respectively. The sensitivity and specificity values for the AUC‐based evaluation were 98.485 and 64.149, respectively, while for the TSS‐based evaluation, the values were 98.485 and 63.847.

### 3.2 Current Habitat Suitability for the Grey Crowned Crane

**FIGURE 2:**
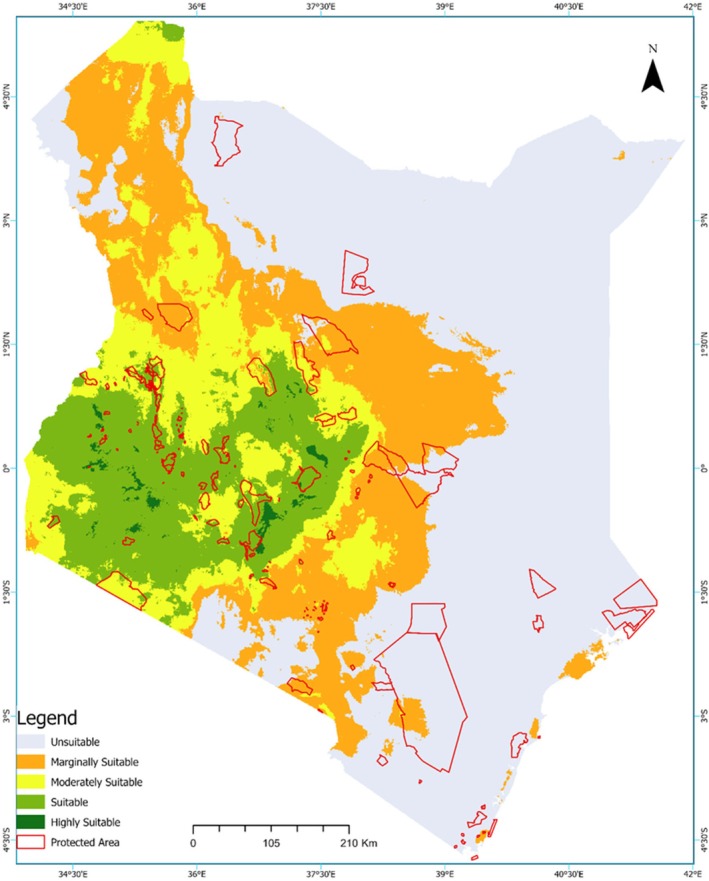
Ensemble model prediction of the current climatic suitability for the Grey Crowned Crane in Kenya. The map shows continuous logistic predictions where dark green areas represent high climatic suitability and light grey areas represent low suitability. The predicted suitability map is overlaid with Kenya's protected areas to illustrate the spatial relationship between suitable crane habitats and existing conservation areas.

The current distribution model, based on occurrence data, produced a continuous predictive map illustrating habitat suitability for Grey Crowned Cranes across Kenya (Figure 2). Highly suitable and suitable habitats (dark and light green zones) are concentrated in the western and southwestern regions, with additional moderately suitable habitats extending into parts of the central region. In contrast, much of the eastern and northeastern regions, as well as the coastal areas, remain marginal to unsuitable (orange and light grey shading), reflecting limited habitat availability for the species.

### 3.3 Variable Importance of Climatic Variables Influencing Habitat Suitability for Grey Crowned Crane

**FIGURE 3:**
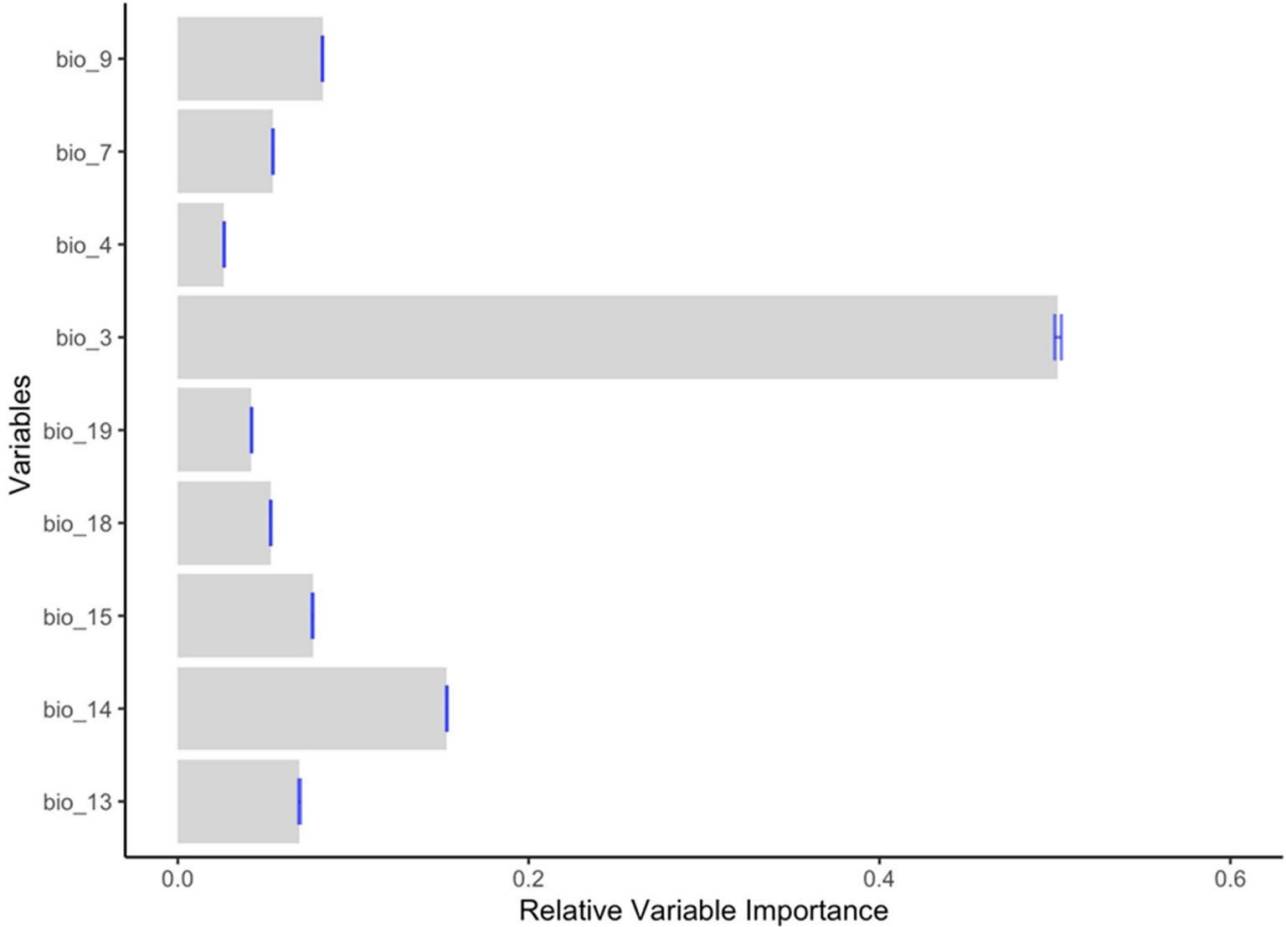
Relative importance of climatic variables to the distribution of Grey Crowned Crane in Kenya.

The analysis of relative variable importance for the Grey Crowned Crane indicated that isothermality (Bio 3) was the most influential predictor, contributing 55.9% to the model. This was followed by precipitation of the driest month (Bio 14; 16.1%), precipitation seasonality (Bio 15; 6.5%), precipitation of the wettest month (Bio 13; 5.4%), and mean temperature of the driest quarter (Bio 9; 5.4%). The remaining variables each contributed less than 5% to the model (Figure 3).

### 3.4 Response Curves

**FIGURE 4:**
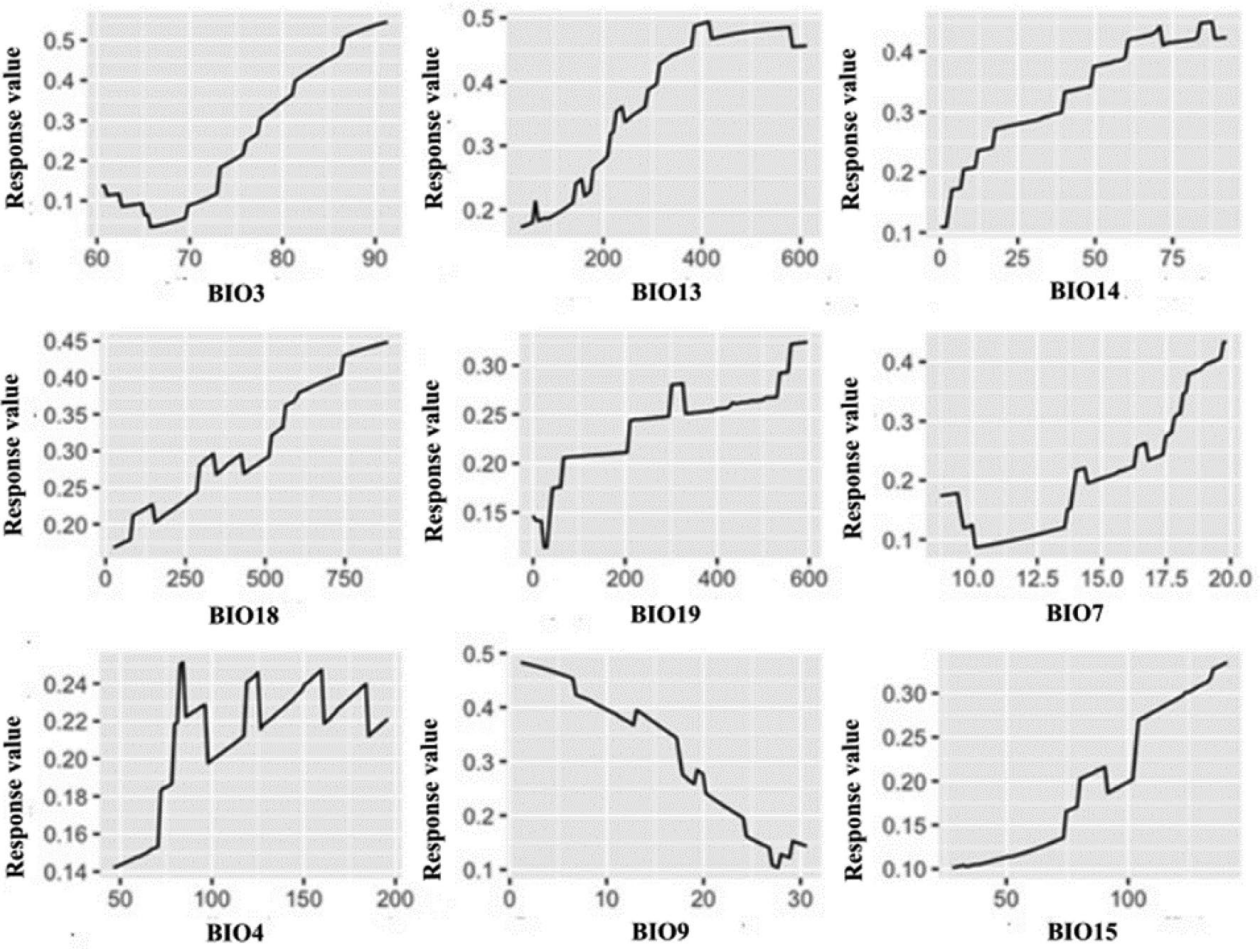
Response curves of 9 bioclimatic variables in Grey Crowned Crane habitat distribution model. Curves show the marginal effect of each predictor on the response value, with all other variables held at their mean values. Bio 3 (Isothermality); Bio 13 (Precipitation of Wettest Month); Bio 14 (Precipitation of Driest month); Bio 18 (Precipitation of Warmest Quarter); Bio 19 (Precipitation of coldest Quarter); Bio7 (Annual temperature range); Bio 4 (Temperature seasonality); Bio 9 (Mean Temperature of Driest Quarter); Bio 15 (Precipitation Seasonality).

The response curves indicate that habitat suitability for Grey Crowned Crane increased steadily with isothermality (°C), with a near linear increase when isothermality values were greater than 50. Precipitation of the wettest month (mm) exhibited a positive relationship, with predicted habitat suitability increasing steadily until it reached a plateau of about 400 mm. Similar positive trends were observed for precipitation of the driest month and precipitation during the warmest and coldest quarters. In contrast, increasing mean temperature during the driest quarter was associated with a decline in predicted habitat suitability. (Figure 4).

### 3.5 Overlap of Grey Crowned Crane Distribution With Protected Areas in Kenya

The national habitat suitability model (Figure 2) revealed that although Kenya has extensive areas suitable for Grey Crowned Cranes, only a small portion of these areas lies within formal protected areas, with the overwhelming majority lying outside such designated zones. The overlay analysis with parks and reserves indicated very limited overlap between protected areas and the most highly suitable zones, suggesting that a large proportion of highly suitable habitats for the Grey Crowned Crane occur outside the current protected area network. Categorically, only 10.11% of the entire protected area was found to be in the highly suitable and suitable habitat areas for cranes. The Masai Mara National Reserve was essentially within moderately suitable habitats, with a smaller portion extending into the suitable category. The Aberdare National Park spanned both suitable and moderately suitable regions, Lake Nakuru National Park was classified as moderately suitable, and Ruma National Park occurred in the suitable zone. Meru National Park was primarily located within marginally suitable habitats, with only a small section overlapping moderately suitable areas, whereas Buffalo Springs National Reserve was situated in moderately suitable regions. Amboseli National Park was situated mainly within marginally suitable habitats, with only a very small section classified as moderately suitable. Tsavo East National Park, along with the northern and eastern arid reserves, was primarily located in marginally suitable or unsuitable areas. Nairobi National Park was predominantly within moderately suitable habitats, with sections extending into marginally suitable areas.

### 3.6 Future Distributions of the Grey Crowned Crane

**FIGURE 5:**
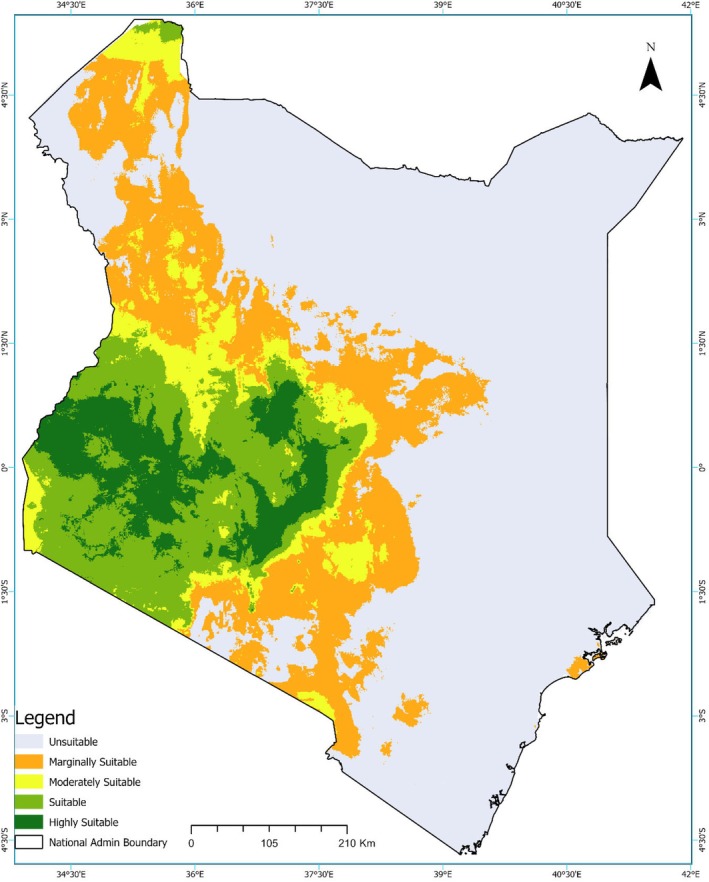
Predicted distribution of Grey Crowned Crane habitat under future climate projections (SSP2‐4.5, middle‐of‐the‐road scenario in 2045). The maps display a continuous logistic prediction, with green areas indicating the highest climatic suitability, while white areas represent unsuitability as derived from the ensemble model.

Under the Shared Socioeconomic Pathway 2‐4.5 (SSP2‐4.5) scenario, projected habitat suitability for the Grey Crowned Crane in Kenya shows a clear redistribution of suitability classes. Areas of high suitability, concentrated in the western and southwestern regions, persist, with smaller patches emerging in the central highlands (Figure 5). Moderately suitable habitats expand across the western, central, and southeastern zones, encompassing areas that previously had lower suitability. In contrast, marginally suitable habitats dominate the eastern and northern regions, indicating reduced habitat quality relative to higher classes. Extensive unsuitable areas remain in the arid and semi‐arid northern and northeastern zones and along much of the coastal strip, reflecting low predicted occurrence probability.

**FIGURE 6:**
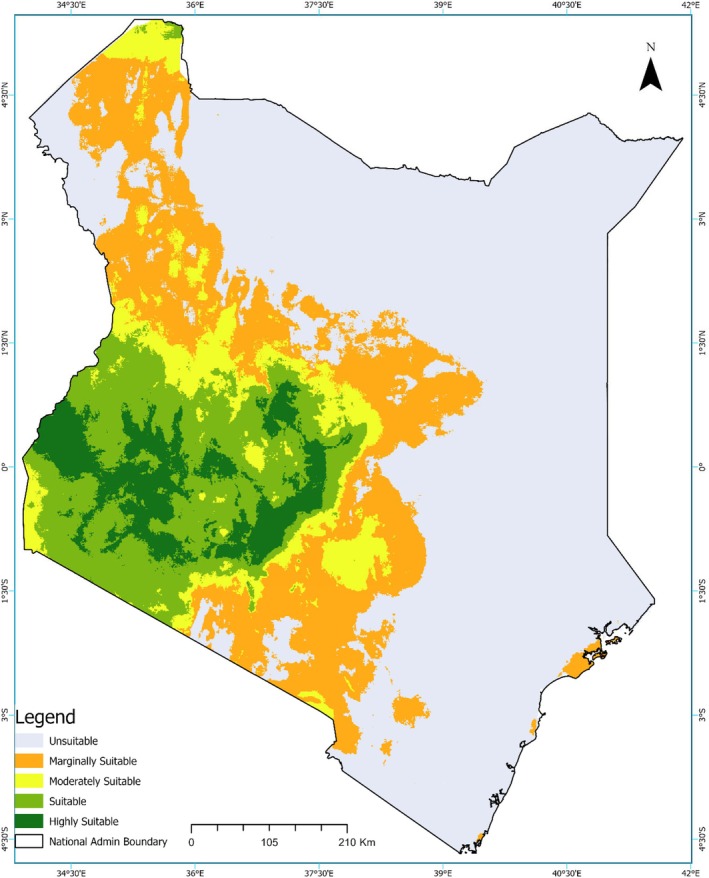
Predicted distribution of Grey Crowned Crane habitat across Kenya under future climate scenario SSP5‐8.5 (fossil fuel development, 2045).

Under the Shared Socioeconomic Pathway 5–8.5 (SSP5‐8.5) scenario, projected habitat suitability for the Grey Crowned Crane in Kenya shows further contraction and redistribution of suitability classes. Areas of high suitability persist mainly in the southwestern and western regions, with smaller patches extending into the southern Rift Valley and central highlands (Figure 6). Moderately suitable habitats occur across the western, central, and southeastern regions, though their extent slightly declines compared to SSP2‐4.5. Much of the eastern and northern regions exhibit only marginal suitability, whereas the arid north and coastal zones remain largely unsuitable, signifying limited potential for the species' presence.

The current baseline scenario estimates 335,335.5 km^2^ of suitable habitat for the Grey Crowned Cranes in Kenya. Here, “suitable habitat” refers to all cells classified as marginally suitable, moderately suitable, suitable, or highly suitable based on the reclassified ensemble model outputs (Section 2.6; Table 2). By 2045, under the SSP2‐4.5 scenario, suitable habitat is projected to decline sharply to 169,263.8 km^2^ (−49.5%), and under the SSP 5–8.5 scenario to 175,908.2 km^2^ (−47.5%) (Table 3). Although overall suitable habitat decreases, some areas classified as highly suitable and suitable show localized increases under both future climate scenarios, indicating potential emerging refugia for the species.

**TABLE 3:** Extent and change in suitable habitat (km^2^ and %) in Kenya for the current baseline and two future climate scenarios (SSP2‐4.5 and SSP5‐8.5) projected for 2045.

Habitat suitability scenario	Area (km^2^)	Change (km^2^)	% change
Current (baseline)	335,335.5	—	—
SSP2‐4.5	169,263.8	−166,071.7	−49.52
SSP5‐8.5	175,908.2	−159,427.3	−47.54

## 4 Discussion

This study provides the first national‐scale prediction of climatically suitable habitats for the Grey Crowned Crane in Kenya and evaluates how these habitats may change under future climate scenarios. The model performed well, indicating strong predictive capacity for identifying potential crane habitats. Our results show that highly suitable habitats are concentrated in western and southwestern Kenya, while eastern and northern regions remain largely unsuitable. Climatic variables related to temperature stability and precipitation patterns were the most influential predictors of habitat suitability. Importantly, the analysis revealed that most suitable habitats occur outside the current protected area network, and future climate projections suggest a substantial decline in suitable habitats across the country. These findings highlight significant conservation challenges for the species and emphasize the need for improved habitat protection and climate‐adaptive conservation strategies.

The ensemble model demonstrated strong predictive performance, with high AUC and moderate TSS values indicating reliable discrimination between suitable and unsuitable habitats. The high AUC (0.908) and moderate TSS value suggest that the selected climatic variables effectively captured environmental conditions associated with Grey Crowned Crane occurrence in Kenya. AUC values above 0.9 are generally considered to indicate excellent model performance, while TSS values above 0.6 reflect good agreement between predicted and observed distributions (Phillips et al. 2006). These results therefore provide confidence in the model's ability to identify areas of suitable habitat and support its application for conservation planning and future climate projections.

The current habitat suitability model indicates that highly suitable habitats for the cranes are concentrated mainly in western and southwestern Kenya, with additional moderately suitable areas extending into parts of the central region. In contrast, much of eastern and northern Kenya, as well as coastal regions, were predicted to be marginally suitable for the species. The concentration of highly suitable habitats in western Kenya likely reflects the region's favorable climatic conditions and the presence of extensive wetlands, floodplains, and moist grasslands, which, according to Morrison et al. (2019), are commonly associated with Grey Crowned Crane occurrence. These landscapes are known to provide suitable ecological conditions for the species, including wetland and grassland environments that support crane populations. In contrast, arid and semi‐arid regions of eastern and northern Kenya experience lower rainfall and limited wetland availability (World Bank 2022), which likely constrains the distribution of this wetland‐dependent species.

Climatic variables played a significant role in shaping the predicted distribution of Grey Crowned Cranes across Kenya. Among these is isothermality (BIO3), which measures the stability of daily relative to annual temperature variation, emerged as the most influential predictor, contributing over 50% to model importance. This suggests that the cranes prefer environments with relatively stable temperature regimes, which likely support wetland persistence, vegetation structure, and contribute to suitable environmental conditions for the species (Deimeke et al. 2013). Precipitation‐related variables, including precipitation of the driest month and precipitation seasonality, also played important roles, highlighting the dependence of crane habitats on reliable rainfall to maintain wetland ecosystems. Wetlands sustained by consistent rainfall provide key food resources such as seeds, insects, and small invertebrates critical to the species' diet (Wamiti 2022; Wachu et al. 2025).

The response curves further emphasize the importance of precipitation and temperature patterns in determining habitat suitability for the Grey Crowned Crane. Habitat suitability increased with increasing precipitation during both the wettest and driest months, indicating that the species prefers areas with relatively consistent water availability throughout the year. Continuous water availability supports the persistence of wetlands and moist grasslands that are commonly associated with suitable crane habitats. Conversely, increasing mean temperature during the driest quarter was associated with declining suitability, suggesting that higher temperatures during dry periods may reduce wetland extent and habitat quality (Rahimi et al. 2020).

An important finding of this study is the spatial mismatch between predicted suitable habitats and the current protected area network in Kenya. The analysis revealed that the majority of highly suitable habitats occur outside formal protected areas, with only a small proportion located within the formal protected areas. This pattern may reflect the historical establishment of many protected areas primarily to conserve large mammals and savannah ecosystems (Western et al. 2009), rather than wetland‐dependent bird species. As a result, many wetlands and agricultural landscapes that provide suitable habitats for Grey Crowned Cranes fall outside protected areas and remain vulnerable to land‐use change, wetland drainage, and agricultural intensification. Similar conservation gaps have been reported globally, where protected areas do not always coincide with the most suitable habitats for threatened species due to historical or socio‐economic factors influencing reserve placement (Rodrigues et al. 2004).

Future climate projections suggest substantial changes in the distribution of suitable habitats for the Grey Crowned Crane in Kenya. Both climate scenarios examined in this study indicate a significant decline in suitable habitats by 2045, with nearly half of the currently suitable area predicted to be lost. In this study, suitable habitat refers to areas classified as marginally suitable, moderately suitable, suitable, or highly suitable based on the reclassified habitat suitability scores. These projected reductions are likely associated with increasing temperatures and shifting rainfall patterns, which are expected to influence wet habitat hydrology and reduce the persistence of suitable habitats across parts of the country (IPCC 2021). Although western Kenya is projected to remain an important refuge for the species, the overall contraction of suitable habitats highlights the potential vulnerability of Geay Crowned Crane populations to climate‐driven habitat changes. However, the projections also indicate localized increases in areas classified as highly suitable and suitable under both future climate scenarios. These increases suggest that some regions may become more environmentally favorable for the species, potentially acting as emerging refugia under future climatic conditions. This pattern may have important implications for conservation planning, as the expansion of highly suitable habitats could provide opportunities for the species to persist if populations are able to utilize these emerging areas. It may also create opportunities for establishing new protected areas in locations projected to become highly suitable in the future, particularly where current land use or policy constraints limit the protection of existing key habitats.

These findings highlight the need for conservation strategies that extend beyond traditional protected areas. Since many suitable habitats occur in agricultural and community‐managed landscapes, conservation efforts should prioritize wetland protection, sustainable land use practices, and community‐based conservation initiatives. Strengthening collaboration between conservation agencies and local communities will be essential to safeguard key habitats for the Grey Crowned Crane. Integrating climate considerations into habitat management and land‐use planning will also be critical to supporting the persistence of crane populations under changing environmental conditions.

## 5 Conclusion

This study provides the first national‐scale assessment of climatically suitable habitats for the Grey Crowned Crane in Kenya and evaluates potential changes under future climate scenarios. The results indicate that climatic factors, particularly isothermality and precipitation patterns, strongly influence the distribution of suitable habitats for the species. Highly suitable areas are concentrated in western and southwestern Kenya, while eastern and northern regions remain largely unsuitable. Future climate projections under both SSP2‐4.5 and SSP5‐8.5 scenarios indicate a substantial contraction of suitable habitats by midcentury, highlighting the potential vulnerability of Grey Crowned Crane populations to climate change. Because many suitable habitats occur outside the protected area network, effective conservation will require integrating wetlands and surrounding landscapes within community‐managed and agricultural areas. Strengthening wetland protection and incorporating climate considerations into conservation planning will be important for supporting the long‐term persistence of the species in Kenya and may also provide useful insights for conservation planning in similar ecological contexts worldwide.

## Author Contributions

**Carolyne M. Wachu:** conceptualization (lead), data curation (lead), formal analysis (lead), investigation (lead), methodology (lead), writing – original draft (lead). **Benson M. Mwangi:** conceptualization (supporting), supervision (equal), writing – review and editing (supporting). **James Jumbe:** conceptualization (supporting), methodology (supporting), supervision (equal), writing – review and editing (supporting). **Wanyoike Wamiti:** conceptualization (supporting), methodology (supporting), supervision (equal), writing – review and editing (supporting). **Denis Kiptoo:** formal analysis (supporting), software (supporting).

## Funding

This work was supported by the International Crane Foundation.

## Conflicts of Interest

The authors declare no conflicts of interest.

## Data Availability

The data supporting the findings of this study are openly available from the Global Biodiversity Information Facility (GBIF), including Grey Crowned Crane occurrence data (https://doi.org/10.15468/dl.ndxqdf), and from WorldClim v2.1 for environmental variables (https://www.worldclim.org/data/worldclim21.html).
